# Dental students' attitudes on cardiopulmonary resuscitation training via virtual reality: an exploratory study

**DOI:** 10.1038/s41415-023-6388-2

**Published:** 2023-10-27

**Authors:** Ruza Bjelovucic, Jesper Bak, Jan Wolff, Pankaj Taneja

**Affiliations:** 41415341337001 PhD Student, Oral and Maxillofacial Surgery and Oral Pathology, Department of Dentistry and Oral Health, Aarhus University, Aarhus, Denmark; 41415341337002 Oral Surgeon, Oral and Maxillofacial Surgery and Oral Pathology, Department of Dentistry and Oral Health, Aarhus University, Aarhus, Denmark; 41415341337003 Professor and Head of Section, Oral and Maxillofacial Surgery and Oral Pathology, Department of Dentistry and Oral Health, Aarhus University, Aarhus, Denmark; 41415341337004 Assistant Professor, Oral and Maxillofacial Surgery and Oral Pathology, Department of Dentistry and Oral Health, Aarhus University, Aarhus, Denmark

## Abstract

**Purpose ** Resuscitation guidelines have advocated the use of virtual learning as a form of pre-course e-learning. Virtual reality (VR) has been identified to provide a method of constructive learning with instant feedback. There are increasing publications of VR use in cardiopulmonary resuscitation (CPR) training; however, there is a dearth from the dental profession. Therefore, the aim of this exploratory study was to investigate dental students' opinions in CPR training using VR.

**Methods** In total, 120 dental students undertook both conventional (manikin) and VR CPR training in a cross-over design. The VR scenario was in a hospital setting. Following, students completed a questionnaire evaluating their experiences.

**Results ** The majority of students (n = 88) reported that this was the first time that they had utilised VR. The experience of using VR in CPR training was rated as very good. Most students felt that the inclusion of VR in CPR training created a better learning experience and had a high learning potential. However, the hospital setting was not entirely relevant.

**Conclusion** Dental students recommended that VR CPR training should be used as an adjunct to conventional training in dental education, but the VR scenario would benefit being a virtual dental environment.

## Key points

- Dental students find cardiopulmonary resuscitation training using virtual reality beneficial.
- This is the first article assessing virtual reality use in cardiopulmonary resuscitation training in dentistry.
- The training context, that is, virtual world, would be more beneficial if tailored to a dental setting.

## Introduction

Dental professionals are at an increasing risk of being presented with medical emergencies due to an ageing patient population and increasing patient co-morbidities, as well as patients on a greater intake of medication.^1^^,^^2^^,^^3^ In an event of cardiac arrest, there is the expectation that dental care professionals are competent in how to manage and provide treatment.^4^ To aid in preparedness, students and professionals are expected to maintain their knowledge and train in the treatment of medical emergencies and undertaking cardiopulmonary resuscitation (CPR). Nonetheless, a lack of skills with regard to basic life support (BLS) has been reported, with an emphasis on the need for further training.^4^^,^^5^^,^^6^^,^^7^^,^^8^

The common approaches in CPR training are face-to-face teaching, utilising manikins, or independent e-learning courses.^9^ However, and more directed to health care professionals, these methods can lack the realism of the environment that a cardiac arrest is likely to be encountered. Furthermore, face-to-face teaching can be costly to run, difficult to access if high in demand, or if meetings are contraindicated;^10^ the latter demonstrated by the recent COVID-19 pandemic.^11^^,^^12^ The current era of digitisation has provided opportunities for the introduction of innovative technologies.^13^

The *European resuscitation guidelines* have advised that health care professionals, which encompasses dental professionals, undertake CPR training of the highest quality.^14^ Suggestions to achieve this include technology enhanced education, such as cognitive aids and feedback devices, as well as gamified learning, for example, via virtual reality (VR).^14^ Technology enhanced education has been reported to improve retention and facilitate competency assessment, particularly in CPR,^14^ thus the European resuscitation council have advocated the use of virtual learning as a form of pre-course e-learning, thereby allowing for a blended learning approach.

VR uses software technology to provide a three-dimensional training environment. This allows the user to be immersed into the virtual scenario, which has been found to provide a more realistic method of teaching than conventional means, building on the users' psychomotor skills.^13^^,^^15^ VR has been identified to provide a method of constructive learning with instant feedback, developing the confidence of operators while at no risk to patients.^13^^,^^16^ These aforementioned factors may contribute to the exponential growth that has occurred in research of utilising VR in healthcare.^17^

Although publications of VR use in CPR training have gained in momentum, there is a lack of representation from the dental profession. Current European guidelines have emphasised that future research areas should investigate optimal training methods and strategies to improve educational efficiency.^14^ However, with a dearth of studies to verify the acceptance of such training methods, incorporation into dental student/professional CPR training and/or curriculum is unlikely. Therefore, the aim of the present exploratory study was to investigate attitudes of VR CPR training during a scheduled CPR training course for dental students.

## Materials and methods

The Institutional Review Board at Aarhus University (Protocol #2021-108) provided ethical approval and the study participants consented to participate in the study and to have their data used as part of the research. The participants consisted of dental students (n = 120) that were recruited during their annual scheduled CPR training course. The course was delivered every day for four consecutive days, with groups of approximately 16 students from each year partaking twice a day.

All students first received a 30-minute presentation on BSL. Following this, groups were divided into halves, with one half first performing face-to-face CPR training using manikins, and the other half undertaking CPR training using a VR scenario. After each group had completed the designated CPR training (approximately one hour), the groups were swapped. Hence, each student group received two different forms of CPR training on a given day.

### Conventional training

A demonstration of adult BSL using a manikin (QCPR Little Anne, Laerdel Medical, Stavanger, Norway), was first provided. The students then each undertook the exercise of BLS on a manikin. Staff members from the department and trained in BLS provided feedback. Students were aware of reaching the correct compression depths via a 'click' sound from the manikin, as well as correct ventilations observed by the manikin chest rising and falling. A software programme (QCPR Training, Laerdel Medical AS, Norway) was utilised to display to students a live graphical representation for the rate and depth of chest compressions, as well as ventilations.

### VR training

The VR training was undertaken in a separate room from the manikin training. VR training consisted of using a headset and handheld controllers (Oculus/Meta Quest 2, California, USA), with the emergency scenario developed by AATE VR, Aarhus, Denmark. In order to familiarise themselves with the VR software and hardware, the students underwent a short tutorial exercise. This consisted of exposing the user to a virtual environment and guiding them through the movements and functions of the hardware by having to complete standardised set tasks, for example, picking up virtual objects, moving around the virtual environment etc. Each task had to be completed before the next task became available and, once completed, indicated that the user had understood the commands required and how to use the controllers. The student would then undertake the VR CPR training exercise. An instructor was present throughout to help with any technical difficulties.

**Fig. 1:**
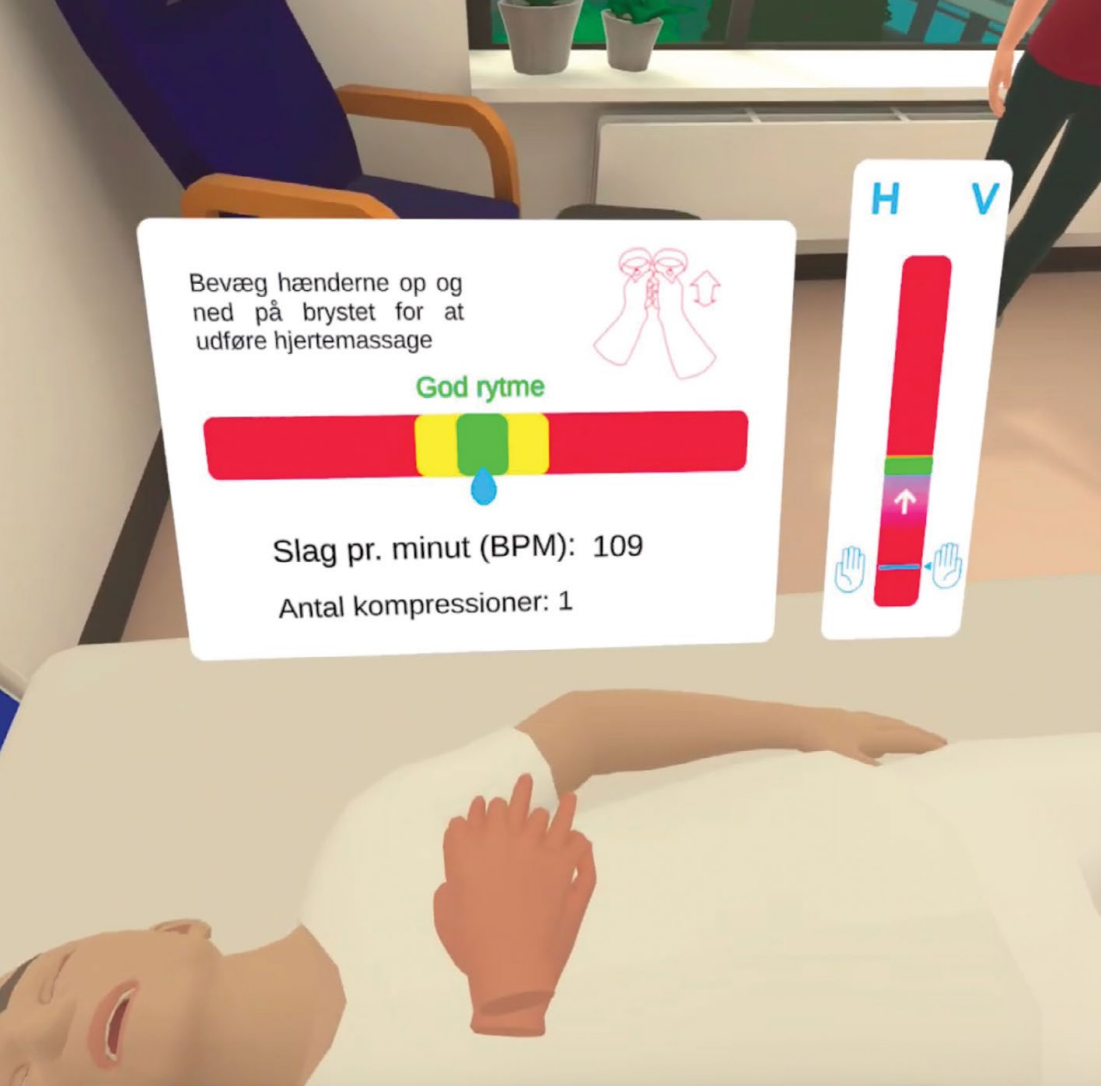
Moving bar and counters displaying beats per minute and number of compressions in the VR CPR scenario. Image reproduced with permission from AATE VR

The students had to perform a number of tasks as per the BSL guidelines/algorithm,^18^ for example, check for responsiveness, call for help, provide chest compressions and ventilations etc. Chest compressions were undertaken by moving the handheld controllers in a vertical axis, while compression depth and rate were automatically measured (Fig. 1). There was no haptic response provided, other than appropriate vibrations in the controller, which matched the actions of the user. Additionally, during the exercise, prompts were displayed that provided questions with a selection of four potential answers, with the scenario continuing once the correct answer was provided. The VR training was completed once the student had finished the VR scenario.

Following the VR training, each student was requested to complete a questionnaire (Appendix 1).

### Questionnaire

The questionnaire was developed based on previous studies that have investigated forms of VR CPR training^19^^,^^20^ and evaluated four aspects: virtual environment and scenario; hardware utilised; educational opinions; and overall experience (see Appendix 1).

Answers to questions were requested via a 7-point Likert scale (LS) for 11 of the questions (Q5-15), a yes/no selection (Q16 and Q17), and free text (Q18).

### Statistical analysis

The results are presented as complete, modal and interquartile values. Normality was evaluated using the Shapiro-Wilk test and analysing Q-Q plots. A sample size calculation was not performed as the study was exploratory. A chi-squared test was used to evaluate differences in age between the semester groups. A two-tailed Mann-Whitney U test was used to compare participant sex between the semesters, as well as to compare the results from questions that utilised a Likert scale. A p-value of <0.05 was determined as statistically significant and a Bonferroni correction was applied for multiple comparisons. All analyses were conducted using SPSS version 28.

## Results

**Fig. 2:**
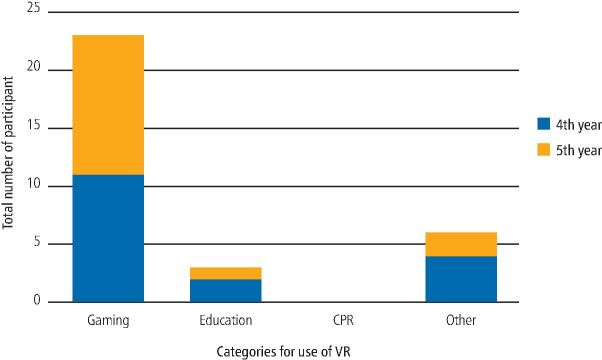
Responses for the categories that participants' had previously used VR

A total of 120 participants, comprising of fourth- (n = 63; mean age = 24.8; range = 22-41) and fifth-year (n = 57; mean age = 25.3; range = 23-30) dental students completed the study. There were no differences in sexes between the year groups (p = 0.795), with the majority consisting of women and the same number of men (n = 12) per group. For most participants (n = 88), this was the first time that they had utilised VR, with no students having used VR previously for CPR training (Fig. 2).

The responses from the year groups were initially collated separately (Table 1, Table 2); however, there were no significant response differences identified (Table 3) and therefore, the results from each year group were combined (Table 4).

**Table 1:** Responses from fourth-year students to questions using a Likert scale

Question	1	2	3	4	5	6	7
5	9	12	22	11	4	4	1
6	16	24	13	5	4	1	0
7	6	17	20	9	6	3	2
8	20	19	18	1	2	3	0
9	21	18	12	5	6	1	0
10	3	3	3	4	2	19	29
11	14	15	15	10	4	4	1
12	25	18	12	4	1	1	2
13	0	1	4	4	6	29	19
14	0	0	6	10	17	18	12
15	28	20	10	1	2	1	1

**Table 2:** Responses from fifth-year students to questions using a Likert scale

Question	1	2	3	4	5	6	7
5	6	19	13	12	3	4	0
6	20	20	10	5	1	1	0
7	7	21	12	7	6	4	0
8	11	28	10	3	5	0	0
9	16	17	10	6	5	3	0
10	1	0	3	2	8	18	25
11	20	12	11	10	4	0	0
12	21	21	7	5	2	1	0
13	1	1	0	3	9	20	23
14	1	0	5	4	20	18	9
15	29	15	9	3	1	0	0

**Table 3:** Mann-Whitney U test for significance between fourth- and fifth-year student answers to questionnaires that utilised a Likert scale

Question	Mean rank fourth year	Mean rank fifth year	Z-score	P-value
5	61.7	59.1	-0.42	0.676
6	63.8	56.8	-1.16	0.248
7	63.3	57.5	-0.94	0.348
8	59.1	62.0	-0.49	0.628
9	58.3	62.9	-0.74	0.461
10	60.3	60.8	-0.09	0.929
11	65.0	55.6	-1.52	0.129
12	60.7	60.3	-0.06	0.949
13	58.1	63.2	-0.86	0.389
14	60.1	60.9	-0.12	0.903
15	62.2	58.6	-0.61	0.545

**Table 4:** Medial and modal results of combined fourth- and fifth-year group answers to questions that utilised a Likert scale

Question	Median (interquartile range)	Mode
5	3 (2-4)	3
6	2 (1-3)	2
7	3 (2-4)	2
8	2 (1-3)	2
9	2 (1-3)	1
10	6 (6-7)	7
11	2 (1-4)	1
12	2 (1-3)	1
13	6 (6-7)	6
14	5 (5-6)	5
15	2 (1-2)	1

### Virtual environment/scenario

Here, LS end-points = totally agree (1) - totally disagree (7). Most students (n = 44) experienced a strong physical presence in the virtual room (Q6: mode = 2). However, a lack of realism was expressed with feeling that the patient was in front of them (Q5: mode = 3). Nonetheless, realism ratings improved once the participants had further interaction with the virtual patient (Q7: mode = 2). Positive scores were given for the user involvement in the resuscitation process (Q8: mode = 2). In general, all participants found it very easy to fulfil the specified tasks in the VR scenario (Q9: mode = 1) (LS endpoints = very easy [1] - very difficult [7]).

### Hardware

Here, LS endpoints = totally agree (1) - totally disagree (7). The majority of students did not consider that the VR CPR equipment posed any difficulties in completing the scenario (Q10: mode = 7) with their own hands corresponding to the same position and orientation as the virtual hands via the controllers (Q11: mode = 1).

### Educational opinions

Most students agreed that the inclusion of VR in CPR training created a better learning experience (Q12: mode = 1) (LS endpoints = totally agree [1] - totally disagree [7]). The latter was reinforced by most participants rating that VR CPR training had a high learning potential (Q13: mode = 6) (LS endpoints = low potential [1] - high potential [7]), with a high tendency to learn something new from the VR experience (Q14: mode = 5) (LS endpoints = to a low degree [1] - to a high degree [7]).

### Overall opinions

The overall experience of using VR in CPR training was rated as very good (Q15: mode = 1) (LS endpoints = really good [1] - really bad [7]). Overall, 95.0% of participants were interested in trying the scenario again (Q16) and 99.2% of participants would recommend the training experience to others (Q17).

In total, 31 participants provided responses on what worked well, or what may need improving (Appendix 2). In 12 cases, the participants gave positive responses, such as 'good experience', 'good learning aid' and 'works well for learning purposes', to name a few. Seven participants responded with comments that addressed the lack of realism when performing compressions, for example, 'no resistance'. Furthermore, the participants noted that it would have been advantageous to perform the CPR scenario in a virtual dental clinic (n = 6).

## Discussion

At present, an increasing number of VR CPR training solutions are becoming available, which may be attributed to their affordability and accessibility.^21^ However, to the best of the authors' knowledge, no VR products have been utilised and evaluated by dental students/professionals in CPR training. Although cardiac events are rare in a dental setting,^4^ methods to optimise the decay of knowledge are required to ensure preparedness for when such an event may occur.^18^ Especially as the invasiveness of procedures undertaken in the speciality, namely delivery of local anaesthesia and oral surgery, may result in medical emergencies that could cause cardiac events.^4^^,^^22^

For the first time, opinions of dental students have been investigated and the results are in good agreement with studies using different user cohorts focusing on the applicability and usability of VR CPR training.^9^^,^^15^^,^^19^ Despite the fact that most students did not have any prior experience with VR, they all managed to adapt effortlessly to the novel technology and immersive learning scenario. They did not perceive that the goggles and controllers hindered the delivery of compressions when undertaking their VR CPR training;^15^ however, the lack of haptic feedback during compressions was expressed as a disadvantage. Nonetheless, there are certain advantages that virtual training captures, for example (and what could be considered unique to VR), providing an emotional realism to CPR training, as well as introducing the prioritisation of task performance while encompassing the use of a number of responses, namely physiological, psychological and social.^23^

The students emphasised the positive effects of immersive learning. More specifically, they found the VR experience engaging and highly interactive, both physically and virtually. It is therefore no surprise that despite age differences between the semester groups, there were no differences in their overall opinions concerning the use of VR in CPR teaching. A possible explanation for these positive results is the implementation of game design elements in the CPR experience. The gaming approach 'gamification' is generally considered to be more enjoyable and interesting among young learners,^24^ promoting a willingness to learn^13^ and, consequently, recognised as a core focal point for innovation in resuscitation education.^25^ In addition, the novelty effect may play a role whereby users tend to enjoy and perform better because of the new technology,^26^^,^^27^ a phenomenon often overlooked in studies focusing on VR CPR training. To overcome this potential effect, longitudinal studies on VR use in training and student opinions should be considered.

A reoccurring comment received from the students was that the hospital environment did not feel relevant. This aspect contributes to the fidelity of the VR training, that is, how much real experiences are reproduced by the system.^9^ It could be argued that since dental students have limited exposure to hospitals, a scenario based in a dental clinical environment would be more relevant. Further support to a more tailored approach in CPR training for dentists is highlighted by the *European resuscitation guidelines* 2021.^4^ This categorises such an event under special circumstances as, firstly, if a patient is on a dental chair, and secondly, if the need for compressions arises, the dental chair is moved to a horizontal position, and a dental stool is placed under the back of the chair to allow for effective compressions.^4^^,^^28^ These aspects could be incorporated into a VR training scenario to potentially enhance the learning experience, as well as maximize confidence. This highlights a novel area of research with regard to how a context more suitable to the learners (for example, dental undergraduates and postgraduates) could aid in the retention and possible reduction in decay in competency.^29^

Advantages of utilising VR technology, as observed in this study, was the ease of use by the students, and that the instructions were provided in the scenario, reducing the need for clinical staff.^13^^,^^16^ This offers the potential for students to be able to train at any given time and without the need for supervision. Furthermore, dental practices could profit from the technology to instil their skills at any given time. The devices are also capable of implementing over-the-air software updates that offer the unique possibility of always being up-to-date concerning new guidelines and techniques.

The present study is associated with some limitations. Namely, the questionnaire used in this study was not validated. In addition is the unequal sex distribution and narrow age range of included participants, limiting generalisability to the older dental student/professional population.

## Conclusion

The supplementation of manikin CPR training with VR CPR training was well-received by dental students. This was especially concerning the learning experience and the hardware utilised. Dental students recommended that VR CPR training should be used as an adjunct to conventional training in their education; however, noted the VR scenario would benefit from being tailored to a dental clinic setting. This is especially important if considering incorporating such methods into a curriculum. It would now be interesting for future studies to investigate how the difference in the virtual environment, for example, dental clinic to hospital, may affect the learning gain, and quality of CPR delivered by dental students/professionals.

**Figure:**
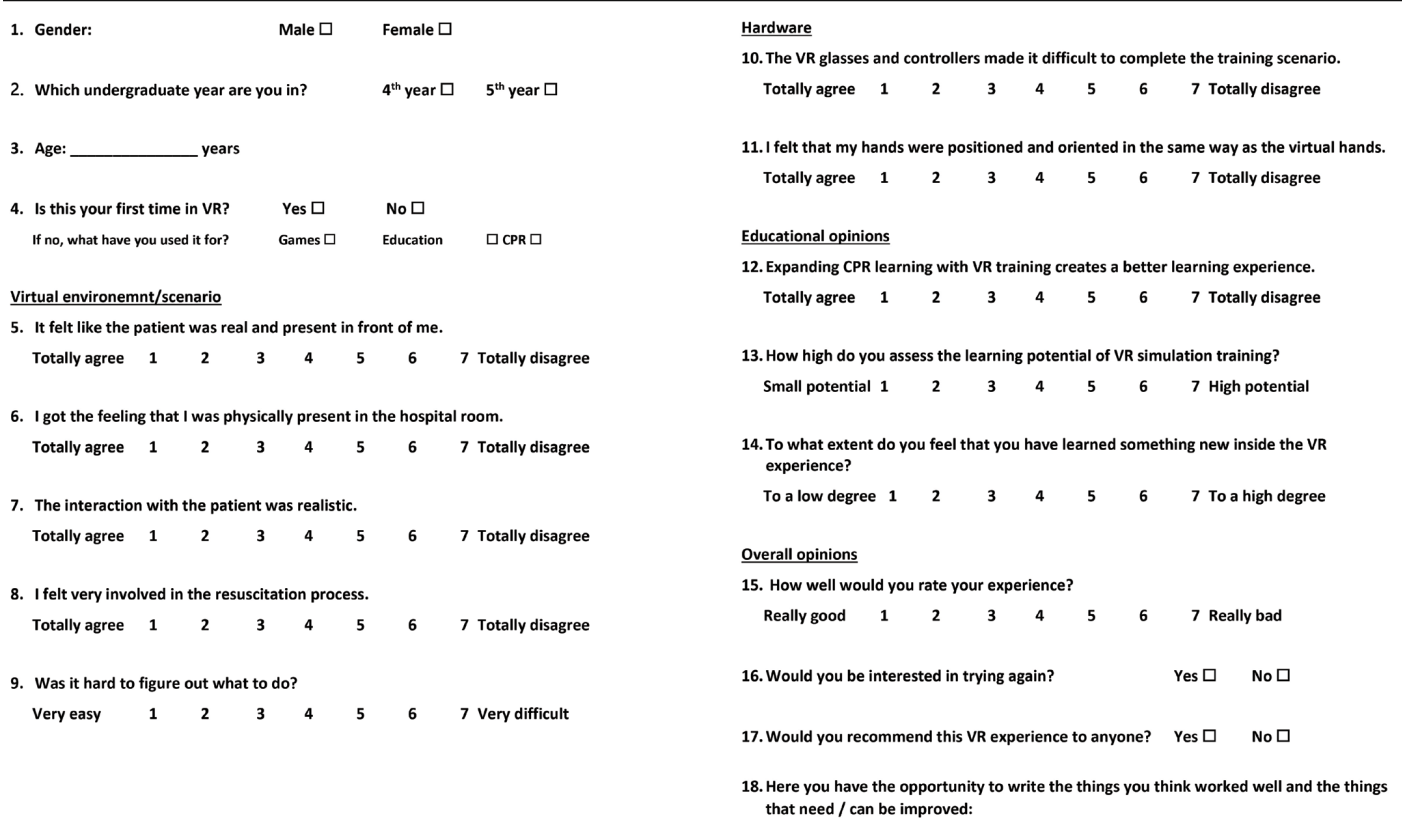
Appendix 1 Questionnaire following VR CPR training

**Figure:**
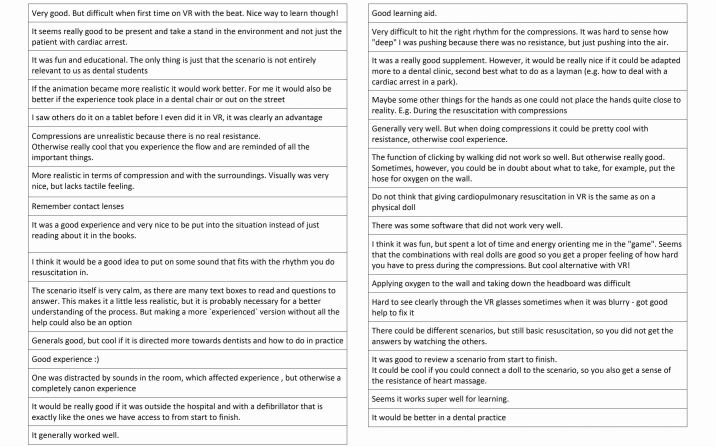
Appendix 2 Free-text responses
